# The role of obesity in carotid plaque instability: interaction with age, gender, and cardiovascular risk factors

**DOI:** 10.1186/s12933-018-0685-0

**Published:** 2018-03-29

**Authors:** Valentina Rovella, Lucia Anemona, Marina Cardellini, Manuel Scimeca, Andrea Saggini, Giuseppe Santeusanio, Elena Bonanno, Manuela Montanaro, Iacopo Maria Legramante, Arnaldo Ippoliti, Nicola Di Daniele, Massimo Federici, Alessandro Mauriello

**Affiliations:** 1 Hypertension and Nephrology Unit, Department of Systems Medicine, University of Rome Tor Vergata, Rome, Italy; 2 Anatomic Pathology, Department of Experimental Medicine and Surgery, University of Rome “Tor Vergata”, Via Montpellier 1, 00133 Rome, Italy; 3 Department of Biomedicine and Prevention, University of Rome “Tor Vergata”, Via Montpellier 1, 00133 Rome, Italy; 4 IRCCS San Raffaele, Via di Val Cannuta 247, 00166 Rome, Italy; 5 OrchideaLab S.r.l, Via del Grecale 6, Morlupo, Rome, RM Italy; 6 Department of Systems Medicine, University of Rome Tor Vergata, Rome, Italy; 7 Vascular Surgery, Department of Biomedicine and Prevention, University of Rome Tor Vergata, Rome, Italy

**Keywords:** Obesity, Stroke, Carotid, Histology, Metabolic syndrome, Age, Gender

## Abstract

**Background:**

In the last decade, several studies have reported an unexpected and seemingly paradoxical inverse correlation between BMI and incidence of cardiovascular diseases. This so called “obesity paradox effect” has been mainly investigated through imaging methods instead of histologic evaluation, which is still the best method to study the instability of carotid plaque. Therefore, the purpose of our study was to evaluate by histology the role of obesity in destabilization of carotid plaques and the interaction with age, gender and other major cerebrovascular risk factors.

**Methods:**

A total of 390 carotid plaques from symptomatic and asymptomatic patients submitted to endarterectomy, for whom complete clinical and laboratory assessment of major cardiovascular risk factors was available, were studied by histology. Patients with a BMI ≥ 30.0 kg/m^2^ were considered as obese. Data were analyzed by multivariate logistic regression and for each variable in the equation the estimated odds ratio (OR) was calculated.

**Results:**

Unstable carotid plaque OR for obese patients with age < 70 years was 5.91 (95% CI 1.17–29.80), thus being the highest OR compared to that of other risk factors. Unstable carotid plaque OR decreased to 4.61 (95% CI 0.54–39.19) in males ≥ 70 years, being only 0.93 (95% CI 0.25–3.52) among women. When obesity featured among metabolic syndrome risk factors, the OR for plaque destabilization was 3.97 (95% CI 1.81–6.22), a significantly higher value compared to OR in non-obese individuals with metabolic syndrome (OR = 1.48; 95% CI 0.86–2.31). Similar results were obtained when assessing the occurrence of acute cerebrovascular symptoms.

**Conclusions:**

Results from our study appear to do not confirm any paradoxical effect of obesity on the carotid artery district. Conversely, obesity is confirmed to be an independent risk factor for carotid plaque destabilization, particularly in males aged < 70 years, significantly increasing such risk among patients with metabolic syndrome.

## Introduction

Obesity is often accompanied by other comorbidities increasing the risk of atherosclerosis, particularly hypertension, dyslipidemia, and type 2 diabetes mellitus, the combination of which is observed in the metabolic syndrome [1, 2]. Despite this mechanistic evidence, the role of obesity as a significant risk factor for development of clinically manifest cardiovascular disease [2–4] is still controversial with both positive and neutral studies [5, 6]. More recently, several studies underlined the so-called “obesity survival paradox” in patients suffering from acute myocardial infarction [2, 7–9]. An analogous paradoxical influence of obesity was also demonstrated in an autopsy study on the aortic district [10].

The influence of obesity on atherosclerotic disease in the carotid district is still unclear. While obesity is regarded as a well-established risk factor for stroke [11–14], some studies reported significantly lower mortality rates in stroke patients with higher body mass index (BMI) values [15–17]. Such conflicting evidence may be due to the fact that, in the carotid district, the atherosclerotic disease has been mainly investigated through imaging methods, instead of histologic evaluation. Nevertheless, histology is regarded as the gold standard for assessing the degree of atherosclerotic plaque vulnerability as well as the presence of cap rupture and acute luminal thrombosis, which is the major source of emboli in the cerebral circulation [18–20]. Furthermore, it remains still unknown whether the pro- or anti-atherosclerotic effect of obesity may be affected by age, gender and other major cerebrovascular risk factors.

The main target for preventing ischemic cerebrovascular events is the identification of vulnerable plaques, prior to the onset of acute clinical symptoms. Thus, the evaluation of any correlation between obesity and plaque histologic features may be of significant clinical relevance. Here we show results from a clinicopathological evaluation of the role of obesity in the destabilization of carotid plaques and development of cerebrovascular events.

## Materials and methods

### Case selection

A total of 390 carotid specimens from the Interinstitutional Carotid Tissue Bank (ICTB) [18] were studied; carotid samples were collected from 265 symptomatic patients (major stroke or transient ischemic attack; 67.9%) and 125 asymptomatic patients (32.1%) underwent to surgical carotid endarterectomy (CEA) at the University of Rome Tor Vergata (Italy).

Among symptomatic patients, only those diagnosed with thrombo-embolism due to carotid atherosclerosis were included, whereas patients referred to any other cause of thrombo-embolism assessed by clinical examination and imaging were excluded. Specifically, cerebral CT scan study and angiographic examination of extra and intracranial carotid arteries and their branches were performed as routine pre-operative evaluation in all patients. Exclusion factors from the study were: (1) a possible cardiac source of embolization (rhythm disorders, recent myocardial infarction, stenosis, prolapse or calcification of mitral valve, left ventricular thrombus, mechanical cardiac valves, atrial myxoma, endocarditis, dilated cardiomyopathy, a patent foramen ovale), (2) stenosis greater than 50% of Willis circle.

All asymptomatic patients showed a carotid stenosis of at least 60%. For each patient, one carotid sample was collected. Informed consent was obtained from all subjects enrolled in this study. Study protocol adhered to ethical guidelines of the 2000 Declaration of Helsinki and was granted an a priori approval by the IRBs of our Institution.

### Risk factors

Patients with a BMI ≥ 30.0 kg/m^2^ were considered as obese, as defined by the American Heart Association (AHA) criteria [13]. Additional cardiovascular risk factors were defined as follows: (a) hypertension: systolic BP ≥ 140 mmHg and/or a diastolic BP ≥ 90 mmHg corresponding to stage 1 of JNC7 [21] and stage 2 of 2017 ACC/AHA guidelines [22]. Any patient under previous treatment with antihypertensive drugs was considered as being included under the diagnosis of hypertension; (b) diabetes mellitus: fasting blood glucose > 126 mg/dL and/or oral glucose-lowering treatment and/or insulin therapy; (c) smoke: patients with tobacco dependence as well as those who had stopped smoking for < 5 years; (d) metabolic syndrome, as defined in the AHA scientific statement [1]. In order to evaluate the dyslipidemia [23] low-density lipoprotein cholesterol (LDL-C) was calculated by the Friedewald equation [24]: LDL-C = cholesterol – [HDL-C + (triglycerides/5)]. A value of LDL-C of > 100 mg/dL was utilized as cut-off between high and low levels. Treatment with statins was also used as a criterion for inclusion in this category.

### Histology

Sampling and histological methods have been previously reported [18, 25, 26]. Intraoperatively, carotid plaques were removed in block, immediately fixed in 10% buffered formalin, transversely cut every 5 mm, paraffin-embedded, and stained with hematoxylin–eosin and Movat stain. The entire plaque was evaluated by histology.

Plaques were classified into two categories according to the modified AHA atherosclerosis classification [27]: stable plaques and unstable plaques. Unstable plaques included those with cap rupture associated with acute thrombosis or with organized thrombi as well as vulnerable plaques or thin-cap fibroatheromas (TCFAs), the latter being characterized by the presence of a fibrous cap < 165 µm-thick and a marked infiltration by CD68 positive macrophages (at least 25/hpf), with no plaque rupture. Stable plaques included fibroatheromatous plaques with thick fibrous caps (> 165 µm) associated with the presence of calcification and a variable necrotic core [28], as well as healed plaques, defined as those showing multilayers of fibrous tissue.

### Statistical analysis

Data were analyzed using SPSS version 16.0 (SPSS Inc, Chicago, Ill) software. Continuous variables were expressed as the mean ± SD. The normal distribution of the data was assessed by the Shapiro–Wilk test. Continuous variables were compared using the independent Student t-test or the Wilcoxon rank sum test. Categorical data were analysed using the Chi square test or the Fisher exact test.

Multivariate analysis using stepwise logistic regression (using the “enter” method for variable selection) was utilized to identify independent risk factors which significantly correlate with the presence of cerebrovascular symptoms (symptomatics vs. asymptomatics) or with an unstable carotid plaque. Multivariate analysis was performed in two models. One model was adjusted for the following parameters: age, gender and all the risk factors associated to metabolic syndrome (hypertension, diabetes, smoking habit, dyslipidemia (LDL-C), obesity. A second model was adjusted for age, gender, smoking habit, high cholesterol levels and metabolic syndrome, with or without obesity.

For each of the independent variables in the model, logistic regression was also used to estimate the coefficient (B), the estimated odds ratio (OR) [exp(B)] and the 95% confidence interval for exp(B) The statistical analyses were performed considering (a) all patients, (b) the male and female subgroups, (c) two cohorts of male patients divided according to the value of the median of age, i.e. 70 years.

A 2-tailed p value < 0.05 was considered statistically significant.

## Results

### Baseline data

As shown in Table 1, the mean age of the study population at the time of CEA was 69.8 ± 7.2 years; 273 (70.0%) were male and 117 female (30.0%); 265 patients (67.9%) suffered ipsilateral major stroke or TIA (31.9%), while 125 (32.1%) were asymptomatic individuals who underwent CEA for high grade carotid stenosis.

**Table 1:** Baseline characteristics of patients

	All cases	Symptomatic patients	Asymptomatic, patients	Symptomatics vs. asymptomatics
	N = 390	N = 265	N = 125	p
Age, *mean (SD)*	69.80 (7.21)	70.11 (7.39)	69.17 (6.80)	0.23
Gender				
M	273 (70.0%)	117 (30.0%)	194 (73.2%)	0.04
F	71 (26.8%)	79 (63.2%)	46 (36.8%)	
Obesity	43 (11.0%)	37 (14.0%)	6 (4.8%)	0.007
Hypertension	271 (69.5%)	198 (74.7%)	73 (58.4%)	0.001
Diabetes	94 (24.1%)	65 (24.5%)	29 (23.2%)	0.77
Smoking habit	227 (58.2%)	152 (57.4%)	75 (60.0%)	0.62
Dyslipidemia (high LDL-C)	257 (65.9%)	185 (69.8%)	72 (57.6%)	0.02
Metabolic syndrome	123 (31.6%)	93 (35.2%)	30 (24.0%)	0.03
Drugs				
Statins	147 (37.7%)	101 (38.1%)	46 (36.8%)	0.80
Diuretics	223 (57.2%)	158 (59.6%)	65 (52.0%)	0.16
Previous myocardial infarction/unstable angina	52 (13.3%)	37 (14.0%)	15 (12.0%)	0.59
Histological type of carotid plaque				
Stable plaques	137 (35.1%)	51 (19.2%)	86 (68.8%)	0.001
Unstable plaques	253 (64.9%)	214 (80.8%)	39 (31.2%)	
Ruptured/thrombotic	123 (45.5%)	123 (45.5%)	0	
TCFA	72 (18.5%)	33 (12.5%)	39 (31.2%)	
Thrombus in organization	58 (14.9%)	58 (14.9%)	0	

All patients had at least one risk factor. Hypertension (in 69.5% of cases) and dyslipidemia (in 65.9% of patients) were the risk factors most frequently observed. In particular, 43 patients (11.0%) showed a BMI > 30 kg/m^2^ (obesity). Metabolic syndrome was observed in 123 cases (31.5%) and in 39 of these obesity constituted one of its components. Treatment with statins and diuretics was administered to 37.7 and 57.2% of patients, respectively. All patients in the post-operative and follow-up periods were treated with aspirin (100 md/die).

**Fig. 1:**
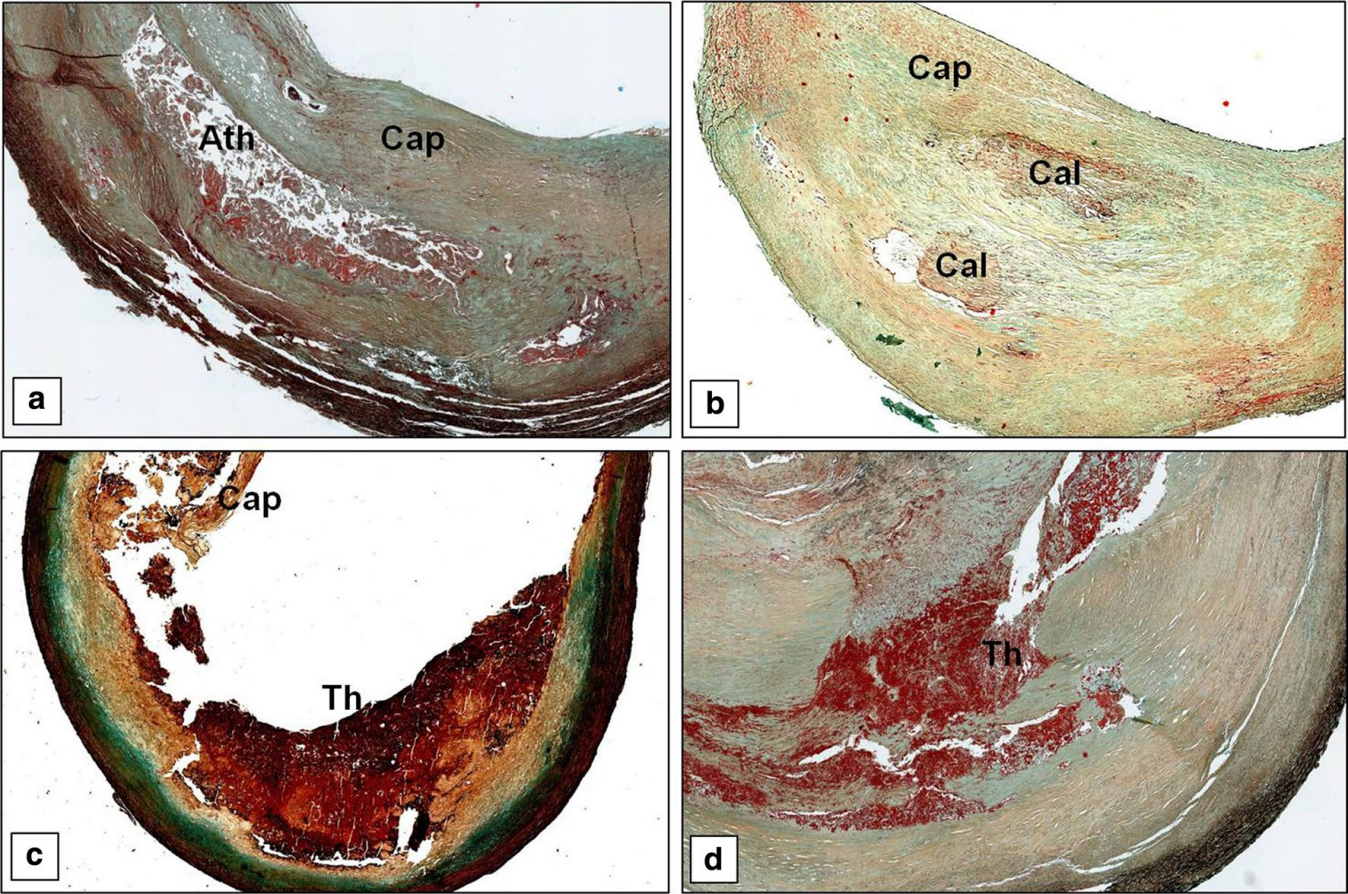
Carotid plaque histology. **a** A stable fibroatheromatous plaque constituted by a large lipidic-necrotic core covered by a thick fibrous cap consisting principally of smooth muscle cells with few macrophages and T lymphocytes (Movat stain, 2×). **b** A stable plaque consisting mainly of fibrous tissue associated to the presence of calcifications (Movat stain, 2×). **c** An unstable ulcerated plaque characterized by a large disruption of the fibrous cap whereby the overlying acute thrombosis is in continuity with the underlying necrotic core (Movat stain, 1.5×). **d** An unstable plaque with an acute thrombus in organization (Movat stain, 4×). *Cap* fibrous cap, *Ath* atheroma or lipid necrotic core, *Cal* calcification, *Th* acute thrombus

The histological examination showed in 253 patients (64.9%) the presence of unstable plaque (with acute thrombosis and/or TCFA), whereas in 137 (35.1%) the presence of the stable one (Fig. 1). Unstable plaques were significantly associated with the presence of symptoms (p = 0.001) (Table 1). The only unstable plaques observed in the asymptomatic group were represented by TCFAs.

### Correlation among risk factors, symptoms and type of plaque

Univariate analysis reported in Table 2 demonstrated that obesity (p = 0.006), gender (p = 0.03), hypertension (p = 0.001) and dyslipidemia (p = 0.01) were the risk factors significantly correlated with the presence of unstable carotid plaques characterized by a high degree of inflammation, thinning and rupture of the cap associated with an acute thrombosis. In addition a significant correlation was also observed between the presence of the risk factors mentioned above and the presence of symptoms (Table 1).

**Table 2:** Correlation between risk factors and type of carotid plaques in all cases

	Unstable plaques	Stable plaques	Univariate analysis	Multivariate analysis	
	N = 253	N = 137	p	p	OR (95% CI)
1st model					
Age, *mean (SD)*	70.0 (7.3)	69.4 (7.0)	0.90	0.20	0.98 (0.95–1.01)
Gender					
M	187 (73.9%)	86 (62.8%)	0.03	0.007	1.96 (1.20–3.18)
F	66 (26.1%)	51 (37.2%)			
Hypertension	200 (79.1.5%)	71 (51.8%)	0.001	0.001	0.29 (0.18–0.47)
Diabetes	63 (24.9%)	31 (22.6%)	0.71	0.97	1.01 (0.59–1.72)
Smoking habit	145 (57.3%)	82 (59.9%)	0.67	0.19	1.37 (0.85–2.20)
Dyslipidemia (high LDL-C)	178 (70.4%)	79 (57.7%)	0.01	0.05	0.63 (0.39–1.01)
Obesity	36 (14.2%)	7 (5.1%)	0.006	0.03	2.58 (1.08–6.19)
2nd model					
Metabolic syndrome	96 (38.1%)	27 (19.7%)	0.001	0.001	2.55 (1.61–4.90)
Metabolic syndrome without obesity	62 (24.5%)	21 (15.3%)	0.006	0.006	1.48 (0.86–2.31)
Metabolic syndrome with obesity	33 (13.0%)	6 (4.4%)	0.001	0.003	3.97 (1.81–6.22)

Multivariate analysis confirmed these results and showed an OR (increased risk of having an unstable plaque) of 2.58 (95% CI 1.08–6.19) for obesity. When the presence of metabolic syndrome was considered in the multivariate analysis, the latter had a highly significant independent effect (p = 0.001) with an OR of 2.55 (95% CI 1.61–4.90). If we considered patients with metabolic syndrome and obesity, the value of OR increased to 3.97 (95% CI 1.81–6.22) compared to the value of 1.48 (95% CI 0.86–2.31) when obesity was not included in the metabolic syndrome (Table 2).

Similar results were obtained when the presence of cerebrovascular clinical symptoms, and not the histological finding of unstable plaques, was considered as the dependent variable in the uni- and multivariate analysis (Table 3). Patients with BMI > 30 and metabolic syndrome were those at highest risk, with an OR of 3.15 (95% CI 1.85–5.10) (Table 3).

**Table 3:** Correlation between risk factors and cerebrovascular symptoms

	Asymptomatic patients	Symptomatic patients	Univariate analysis	Multivariate analysis	
	N = 125	N = 265	p	p	OR (95% CI)
1st model					
Age, *mean (SD)*	70.0 (7.3)	69.4 (7.0)	0.90	0.11	1.03 (0.99–1.06)
Gender					
M	79 (63.2%)	194 (73.2%)	0.06	0.02	0.57 (0.35–0.92)
F	46 (36.8%)	71 (28.8%)			
Hypertension	73 (58.4%)	198 (74.7%)	0.001	0.004	1.98 (1.24–3.17)
Diabetes	29 (23.2%)	65 (24.5%)	0.80	0.86	0.96 (0.56–1.62)
Smoking habit	75 (60.0%)	152 (57.4%)	0.66	0.31	0.78 (0.49–1.25)
Dyslipidemia (high LDL-C)	72 (57.6%)	185 (69.8%)	0.02	0.04	1.64 (1.03–2.61)
Obesity	6 (4.8%)	37 (14.0%)	0.006	0.02	0.34 (0.14–0.86)
2nd model					
Metabolic syndrome	30 (24.0%)	93 (35.2%)	0.03	0.04	1.71 (1.05–3.04)
Metabolic syndrome without obesity	24 (20.2%)	61 (26.3%)	0.24	0.26	1.17 (0.71–1.88)
Metabolic syndrome with obesity	6 (5.9%)	33 (16.2%)	0.01	0.01	3.15 (1.85–5.10)

### Correlation among risk factors, gender and type of plaque

A separate analysis was performed in male and female patients, as reported in Table 4.

**Table 4:** Correlation between risk factors and type of carotid plaques in male and female patients

	Male patients				Female patients			
	Unstable plaques	Stable plaques	Multivariate analysis		Unstable plaques	Stable plaques	Multivariate analysis	
	N = 187	N = 86	p	OR (95% CI)	N = 66	N = 51	p	OR (95% CI)
Age, *mean (SD)*	70.4 (7.2)	68.8 (6.9)	0.03	0.96 (0.92–1.00)	70.5 (7.31)	69.7 (7.44)	0.42	1.02 (0.97–1.08)
Hypertension	146 (78.1%)	43 (50.0%)	0.001	0.27 (0.15–0.49)	54 (81.8%)	28 (54.9%)	0.005	0.29 (0.12–0.69)
Diabetes	45 (24.1%)	20 (23.3%)	0.68	1.15 (0.59–2.23)	18 (27.3%)	11 (21.6%)	0.65	0.81 (0.32–2.04)
Smoking habit	117 (62.6%)	58 (67.4%)	0.19	1.50 (0.82–2.75)	28 (42.9%)	24 (47.1%)	0.54	1.20 (0.58–2.85)
Dyslipidemia (high LDL-C)	128 (68.4%)	46 (53.5%)	0.06	0.58 (0.32–1.07)	50 (75.8%)	33 (64.7%)	0.43	0.71 (0.30–1.66)
Obesity	28 (15.0%)	3 (3.5%)	0.01	5.06 (1.42–18.11)	8 (12.1%)	4 (7.8%)	0.92	0.93 (0.25–3.52)

For male patients the risk factors significantly associated with the presence of unstable carotid plaques were: age (p = 0.03), hypertension (p = 0.001) and obesity (p = 0.01). However, obesity showed the higher OR (= 5.06, 95% CI 1.42–18.11) as compared to those of other risk factors.

In the female patients obesity was not significantly associated to the presence of unstable plaques (p = 0.92). In this subgroup of patients only hypertension was predictive of carotid plaque instability (p = 0.005).

Since in male patients age was an independent risk factor for carotid plaque instability, a different analysis was performed considering (a) male patients with < 70 years and (b) those with ≥ 70 years, value corresponding to the median value of age (Table 5). In patients aged < 70 years obesity showed the highest value of OR observed in all the analyses performed, with a value of 5.91 (95% CI 1.17–29.80). In aged patients (≥ 70 years) the value of OR decreased to 4.61 (95% CI 0.54–39.19).

**Table 5:** Effect of age in the correlation between risk factors, gender and type of plaques

	Male patients < 70 years				Male patients ≥ 70 years			
	Unstable plaques	Stable plaques	Multivariate analysis		Unstable plaques	Stable plaques	Multivariate analysis	
	N = 84	N = 48	p	OR (95% CI)	N = 103	N = 38	p	OR (95% CI)
Hypertension	67 (79.8%)	25 (52.1%)	0.02	0.27 (0.12–0.63)	79 (76.7%)	18 (47.4%)	0.002	0.27 (0.12–0.62)
Diabetes	24 (28.6%)	14 (29.2%)	0.52	1.34 (0.55–3.30)	21 (20.4%)	6 (15.8%)	0.90	0.93 (0.32–2.71)
Smoking habit	63 (75.0%)	34 (70.8%)	0.88	1.07 (0.44–2.59)	54 (52.4%)	24 (63.2%)	0.12	1.97 (0.84–4.58)
Dyslipidemia (high LDL-C)	65 (77.4%)	26 (54.2%)	0.04	0.42 (0.18–0.95)	63 (61.2%)	20 (52.6%)	0.86	4.57 (0.38–1.97)
Obesity	16 (19.0%)	2 (4.2%)	0.03	5.91 (1.17–29.80)	12 (11.7%)	1 (2.6%)	0.16	4.61 (0.54–39.19)

## Discussion

Results reported in this study support the role of obesity as an independent risk factor for carotid plaque destabilization among male patients < 70 years-old, while in older patients and women the effect of obesity appears to be less consistent. Indeed, among patients with BMI > 30 g/m^2^ and age < 70 years, OR for unstable carotid was 5.91 (95% CI 1.17–29.80), the highest OR compared to that of other risk factors. OR for unstable carotid plaque decreased to 4.61 (95% CI 0.54–39.19) in males ≥ 70 years (Table 5), whereas it resulted 0.93 (95% CI 0.25–3.52) in the women group (Table 4).

Various clinical studies reported a significant association between obesity and risk of stroke, independently from the presence of other known cardiovascular risk factors [11–14]. Indeed, patients with a BMI in the 25–50 kg/m^2^ range were shown in the Prospective Studies Collaboration (involving 900,000 patients) to have a 40% increased mortality from stroke [29]. Accordingly, the AHA recommends weight reduction in overweight (BMI = 25–29 kg/m^2^) and obese (BMI > 30 kg/m^2^) individuals, in order to reduce the risk of stroke [13].

The present study not only assessed the correlation between obesity and the occurrence of cerebrovascular symptoms, but also evaluated any association of obesity with the degree of atherosclerotic changes within carotids through histologic analysis of carotid plaques. Importantly, one of the targets of stroke prevention is the identification of plaques at high risk for rupture and thrombosis, i.e. so-called “vulnerable or unstable plaques” [18, 27]. Anatomic and clinical studies have demonstrated that acute cerebrovascular events are pathogenetically related to thrombosis and rupture of vulnerable atherosclerotic plaques, rather than to severity of stenosis [18, 27, 30]. To this end, many efforts have been recently made using non-invasive techniques aimed at identifying high risk plaques prone to rupture and thrombosis [31–34].

Results from our study support the negative impact of obesity in influencing other known risk factors for cardiovascular disease, increasing the risk of cerebrovascular disease and carotid plaque instability among patients with metabolic syndrome. As shown in Table 2, when obesity featured among metabolic syndrome risk factors, OR for plaque destabilization was 3.97 (95% CI 1.81–6.22), a significantly higher value compared to OR in non-obese individuals with metabolic syndrome (= 1.48, 95% CI 0.86–2.31). Similar results were obtained when assessing the occurrence of acute cerebrovascular symptoms (Table 3).

The observation that obesity represents an independent risk factor of plaque vulnerability only in male patients as supported by histological analysis corroborates data from both a recent large meta-analysis [35] and a duplex ultrasound study performed on 1686 patients with cerebrovascular disease [36]. Additionally, in another previous histological study performed on 457 CEA specimens from symptomatic and asymptomatic patients, our group had already observed that women had a significant lower prevalence of thrombotic plaques, as well as smaller necrotic core and hemorrhage extension [37]. Gender-related differences in cardio- and cerebrovascular disease have been extensively demonstrated [36, 38–40]. Sex hormones seem to favor plaque stabilization, through their influence on endothelial function, lipid homeostasis, and inflammation. Several authors have hypothesized an age-dependent effect of estrogens, with a significant reduction of plaque inflammation being observed only in younger patients [38, 41]. Notwithstanding, results of this as well as previous studies from our group [37] appear to show that also among post-menopausal women estrogens seem to have a stabilizing and protective effect on carotid arteries, as demonstrated by a significant lower number of inflammatory cells including macrophagic foam cells within the plaque cap of females as compared to men.

### Obesity and incidence of cardio and cerebrovascular diseases

In the last decade, several studies have reported an unexpected and seemingly paradoxical inverse correlation between BMI and incidence of cardiovascular disease. Extensive clinical data from patients with congestive heart failure and ischemic heart disease seem to support the hypothesis that overweight and obese individuals benefit from a more favorable short- and long-term prognosis [2, 7–9]. These results have been corroborated by autopsy-related and histologic data revealing a lower incidence of coronary atherosclerosis in obese decedents [42, 43]. According to Brodsky et al. [10] morbid obesity seems to exert a protective effect also against developing severe aortic atherosclerosis, even if the pathogenetic mechanisms for such observation are yet to be explained.

On the contrary, other clinical studies failed to show the protective effects of obesity. The LEADER trial demonstrated a prevalence and cardiometabolic impact of obesity in cardiovascular high-risk patients with type 2 diabetes mellitus [44]. In addition, the Partners registry has demonstrated that compared to normal BMI there was an increased burden of CAD for BMI > 25 kg/m^2^ [45]. According to these data, the results of our study do not seem to confirm such paradoxical effect of obesity on the cerebral circulation, especially in the cohort of male patients with an age < 70 years; additionally, a neutral influence seems to exist among female patients (Table 4).

The discrepancy between our results and those obtained with a similar methodology in other vascular districts could be due to the fact that our study did not include the general population, but was rather limited to patients undergoing CEA due to symptomatic disease or presence of a carotid stenosis > 60%. Accordingly, our study did not evaluate the absolute incidence of atherosclerotic disease, but rather was focused on the unstable atherosclerotic plaque since it represents the target for prevention studies.

### Obesity and aging

Another significant clinical result from our study is the observation that risk of carotid plaque destabilization in obese patients is linked to age. Indeed, OR was highest in male patients aged < 70 years. It was not possible to evaluate whether the risk increased in even younger patients as in our cases only 38 individuals were younger than 60 years. Despite this limitation, our results confirm data from other clinical studies reporting an inverse age-dependent association of BMI with all-cause mortality risk, including stroke mortality [14, 46]. In this context, the Global BMI Mortality Collaboration study recently reported a detailed analysis about the correlation between BMI and all-cause mortality, including atherosclerotic disease, showing that BMI was greater in younger than older people and in men than women [47]. Furthermore, prevention of hypertension, obesity, and diabetes in patients aged 44–56 years may substantially prolong heart failure-free survival and decrease heart failure-related morbidity [48, 49].

### Study limitations

Another possible limitation of our study is the criterion used to define obesity, since BMI, waist-to-hip ratio, or waist circumference have been employed to evaluate the presence of obesity in different studies. Data in the literature in this regard are conflicting. According to some authors, abdominal body fat might represent a stronger predictor of stroke risk than BMI [50]. Other studies reported that only BMI was significantly associated with stroke in male patients, whereas for female individuals the ideal criterion to define obesity appeared to be waist-to-hip ratio [51]. In our study the AHA definition of obesity was used [13], as we believe that BMI appears to be the most frequent anthropometric index employed to measure the obesity in the available literature [52, 53].

Moreover, mixing the whole patient population (symptomatic and asymptomatic) may have determined a bias in the results, since patients who already had a stroke also presented a different risk factor profile overall. It was not possible to perform separately the multivariate statistical analysis in the group of symptomatic and asymptomatic patients as the latter was only constituted of 125 cases with only 6 obese, 4 with TCFA and 2 with stable plaques. To overcome this limitation we evaluated the possible correlation of obesity with the instability of the carotid plaque which represents the true target for stroke prevention.

## Conclusions

In sum, the results from our study appear to do not confirm any paradoxical effect of obesity on the carotid artery district. Conversely, obesity is confirmed to be an independent risk factor for carotid plaque destabilization, particularly in males aged < 70 years, significantly increasing such risk among patients with metabolic syndrome.

Further studies are required to assess the clinical implications of our data. We may speculate, however, that gender, along with age and type of atherosclerotic lesions (as assessed by imaging techniques) should be taken into account for a precise risk-stratification of obese patients.

## Abbreviations

BMI: body mass index
CEA: carotid endarterectomy
AHA: American Heart Association
OR: estimated odds ratio
95% CI: 95% confidence interval
TCFA: thin-cap fibroatheroma
